# Preliminary Investigation on Salivary Enzymes of Massese Sheep

**DOI:** 10.3390/ani16071008

**Published:** 2026-03-25

**Authors:** Simona Sagona, Alessia Di Rosso, Francesca Coppola, Chiara Benedetta Boni, Claudia Russo, Lionella Palego, Laura Betti, Gino Giannaccini, Antonio Felicioli, Lucia Casini

**Affiliations:** 1 Department of Veterinary Sciences, Pisa University, Viale delle Piagge 2, 56124 Pisa, Italy; alessia.dirosso@vet.unipi.it (A.D.R.); francesca.coppola@vet.unipi.it (F.C.); chiara.benedetta.boni@phd.unipi.it (C.B.B.); claudia.russo@unipi.it (C.R.); antonio.felicioli@unipi.it (A.F.); lucia.casini@unipi.it (L.C.); 2 Department of Pharmacy, Pisa University, Via Bonanno 6, 56126 Pisa, Italy; laura.betti@unipi.it (L.B.); gino.giannaccini@unipi.it (G.G.); 3 Department of Clinical and Experimental Medicine, Pisa University, Via Savi 10, 56126 Pisa, Italy; lionella.palego@unipi.it

**Keywords:** enzyme activity, saliva, digestion, sheep, welfare, saliva collection method, rumen

## Abstract

Saliva is an important biological matrix that allows the investigation of various welfare parameters; in ruminants, it is abundant and can be easily collected without requiring professional veterinary intervention. The aim of the investigation was to provide additional information on both digestive and antioxidant enzymes of sheep farmed in good welfare conditions, assessed with the Animal welfare indicators (AWIN) protocol. Small surgical forceps holding a Salivette^®^ device (Sarstedt, Nümbrecht, Germany) were gently inserted into the sheep’s mouths without any force. The sheep chewed the swabs independently for a few seconds, allowing saliva collection. Seventeen enzymes from the saliva of 15 sheep were analyzed: antioxidant enzymes (catalase, glutathione S transferase), proteolytic enzymes (trypsin, chymotrypsin, N-aminopeptidases, carboxypeptidase A and B), carbohydrases (glucose oxidase, amylase, cellulase, lignin peroxidase, chitinase and α-glycosidase), and esterases (alkaline and acidic phosphatases, lipase and esterase). Esterase activity showed the highest value (12.95 ± 1.25 U/mg of proteins), whereas lignin peroxidase activity showed the lowest (2.23 ± 0.37 µU/mg of proteins). The activity of all enzymes was observed except for glutathione S transferase and α-glycosidase. Among the enzymes, lipase activity has already been identified as a biomarker of stress in sheep saliva. This investigation may represent a basis for further investigations into the diet and adaptive responses of sheep to different environmental conditions. Furthermore, samples collected using the Salivette^®^ device can be easily obtained without requiring specialized staff and without causing any stress to the animals. Further investigations into the origin of individual enzymes using a proteomic approach are desirable.

## Simple Summary

Saliva is an important biological matrix that allows the investigation of many animal welfare parameters. The aim of this study was to investigate enzyme activity in sheep saliva, providing additional information on both digestive and antioxidant enzymes of sheep farmed in good welfare conditions. Seventeen enzymes were analyzed, grouped into four categories: antioxidant enzymes, proteolytic enzymes, carbohydrases, and esterases. Esterase activity showed the highest value compared to the other enzyme activities, whereas lignin peroxidase activity was the lowest. Glutathione S transferase and α-glycosidase activities were investigated but were not detected in the analyzed samples. This study characterizes the enzymes present in the saliva of healthy sheep, providing new information on ovine physiology. It may also serve as a valuable foundation for future research on sheep adaptive responses to dietary changes and environmental stressors, potentially contributing to the evaluation of health and nutritional status in this species.

## 1. Introduction

Sheep (*Ovis aries*) is a small ruminant species whose products—meat, milk, and wool—are of considerable economic importance in Mediterranean regions and developing countries. This species possesses a highly complex and specialized digestive system, adapted to the efficient utilization of fibrous plant material [1]. Traditionally, in Mediterranean regions, small ruminants are farmed under extensive and semi-extensive systems; however, intensive systems are increasingly being adopted in France, Greece, Italy, Spain and Turkey [2]. In these systems, animals are raised primarily on pasture and receive supplements, especially during winter, such as hay, grains, and minerals, depending on grass availability and the physiological status of the animals [2]. Several authors have suggested that grazing ruminants tend to select a diet that allows the rumen to remain in a state of homeostasis and adaptation, maintaining optimal conditions or at least conditions within an acceptable range [3,4].

The rumen is the largest compartment of the four-chambered stomach typical of ruminants, where microbial fermentation of feed, mainly fibrous components, takes place due to a large and diverse population of microorganisms, including protozoa, bacteria and fungi [5,6].

Microbial activity within the rumen is greatly affected by changes in the ruminal environment [7]. This is particularly important in sheep, as the supply of energy and protein to the small intestine depends largely on the activity of these microorganisms [8]. It has been hypothesized that rumen conditions associated with the fermentation of rapidly fermentable substrates, the increased acidity and osmolality, and the hydrolysis of rapidly degradable proteins, which lead to high ammonia concentrations, may significantly influence diet selection in ruminants [8].

Enzymes derived from ruminal microorganisms are a significant component of salivary fluid due to rumination activity [9]. Saliva plays an important role in the digestive process of ruminants. In ruminants, saliva is secreted by three main glands: sublingual, submaxillary and parotid. The sublingual and submandibular glands produce saliva rich in mucins but lacking significant ash content and alkalinity; their primary function is to lubricate the food bolus. In contrast, the parotid gland secretes a solution containing sodium, potassium, bicarbonate, phosphate and small amounts of chloride during feeding [10]. Parotid saliva does not contain ptyalin but exhibits high alkalinity and strong buffering capacity [9]. Submaxillary, sublingual, and labial saliva contain much lower concentrations of Na^+^, HCO^3−^ and H_2_PO_4_^2−^ compared to parotid saliva [10]. Regarding the enzymatic activity of saliva, a moderate level of acid phosphatase activity, marked alkaline phosphatase activity, low esterase activity and the presence of a pseudocholinesterase activity have been reported in sheep submaxillary fluid [11]. Sheep parotid saliva has also been analyzed using a proteomic approach, and the presence of catalase, carbonic anhydrase, deoxyribonuclease and cathepsin H was observed, while glutathione S transferase was identified in goat saliva [12]. Furthermore, phosphoglycerate mutase 1 and triosephosphate isomerase were also detected in the saliva of sheep [13].

In small ruminants, saliva is a valid biological matrix for the evaluation of stress levels using different markers. By investigating the activity of butyrylcholinesterase, total esterase, adenosine deaminase, and lipase, Contreras-Aguilar and colleagues [14] observed that lipase activity showed significant differences between a control group and groups subjected to two types of stress (i.e., exposure to a dog and isolation). In small ruminants, such as sheep, the biological samples most frequently used for biochemical analyses of welfare evaluation are blood, wool, urine and saliva [15,16,17,18]. However, the first three matrices each present limitations: wool can be used to detect indicators of chronic stress [17], whereas both urine and blood samples require collection by trained veterinary technicians [18]. Conversely, saliva offers several advantages compared to other matrices, as it can be collected easily and non-invasively, even by individuals with limited training [19,20]. Furthermore, ruminants produce large quantities of saliva, ranging from 6 to 16 L/individual per day in sheep [10].

The aim of this work was to investigate a broad spectrum of saliva enzymatic activities in sheep farmed under verified welfare conditions according to the Animal welfare indicators (AWIN) protocol [21]. The enzymes were mainly selected based on the feed characteristics of sheep and antioxidant activity. Seventeen enzymes were investigated and grouped into the following categories: antioxidant enzymes (catalase, glutathione S transferase), proteolytic enzymes (trypsin, chymotrypsin, N-aminopeptidases, carboxypeptidase A and B), carbohydrases (glucose oxidase, amylase, cellulase, lignin peroxidase, chitinase and α-glycosidase), and esterases (alkaline and acidic phosphatases, lipase and esterase).

## 2. Materials and Methods

The experiment consisted of collecting saliva from sheep of the same breed, age, physiological condition and diet. For this purpose, fifteen non-pregnant 2–3-year-old female Massese sheep were randomly selected from a single flock on a farm located in Tuscany, Sant’Ermo (Casciana Terme-Lari), Pisa, Italy (43.5374789, 10.5653075). This area is characterized by a highly anthropic fragmented agro-ecosystem in which small woody areas, interspersed with agricultural and urban zones, are interconnected by a dense network of ecological paths. All the experimental sheep showed no evident signs of clinical diseases (i.e., nasal discharge, diarrhea, mastitis). The sheep were managed under a semi-extensive system and fed on pastures composed of *Avena sativa*, *Hedysarum coronarium*, *Trifolium alexandrinum*, and Poaceae. The animals were provided with the same fodder (hay harvested from summer pastures consisting of *Avena sativa*, *Hedysarum coronarium*, *Trifolium alexandrinum*, and Poaceae), produced by the farmer. Hay was fed *ad libitum* once a day when the animals were stabled. Additionally, during milking, all the animals were fed oats twice a day.

Two saliva samples were collected from the same individuals on two different days, with one sample obtained per day, within two hours after the milking process. Saliva was collected using Salivette^®^ devices (Sarsdedt, Nümbrecht, Germany). Small surgical forceps were used to hold the cotton swab. During sampling, one operator restrained the sheep while a second operator gently inserted the Salivette^®^ into the animal’s mouth without applying force. The sheep independently chewed the swab for 20–30 s, allowing saliva sampling collection, and, subsequently, the swab was enclosed in the provided plastic tube for conservation. To prevent sample degradation, the Salivette^®^ devices were kept in an isothermal box with ice packs and transported to the laboratory within 3 h of collection. The tubes were centrifuged at 3500 rpm for 20 min using a PK130 centrifuge (ALC International Srl, Milano, Italy) to extract the saliva from the cotton swabs. The recovered saliva was aliquoted and stored at −20 °C until analysis.

### 2.1. Welfare Assessment

The AWIN protocol for sheep [21] was used to evaluate the welfare status of the animals investigated. The number of animals to be individually assessed (n = 41) was specified in the AWIN protocol based on the number of adult ewes (n = 120). According to the protocol, two questionnaires were filled out to assess the status of the farm and the sheep: one concerned the number of animals, the characteristics of the farm and management practices, while the other, filled out by an external operator, included observations of the flock, information on health, the human–animal relationship and the quality of resources (water availability, stocking density), as listed in Table 1. For ewes with lambs, space availability was considered satisfactory when it provided at least 2 m^2^ per animal.

### 2.2. Enzymatic Assays

All reagents were purchased from Sigma-Aldrich (Milan, Italy). Spectrophotometric analyses were performed using a Multiskan FC reader (Thermo Scientific, Waltham, MA, USA) and a Lambda 25 UV/VIS spectrometer (PerkinElmer, Waltham, MA, USA).

The protein content of salivary samples was determined according to the Bradford method [22] using γ-globulin as the standard. For all analyses, each sample was assayed in duplicate.

#### 2.2.1. Antioxidant Enzymes

Catalase activity was determined using the method described by Góth [23]. Absorbance was measured at 405 nm, based on the formation of a yellow molybdate–H_2_O_2_ complex, against a no enzyme/no H_2_O_2_ blank. The results were expressed as U/mg of proteins. Glutathione S-transferase activity was measured using a slightly modified procedure described by Habig and colleagues [24]. Briefly, a reaction mixture containing 150 µL of 100 mM phosphate buffer, pH 6.5, 6.5 µL of 1 mM 1-chloro-2,4-dinitrobenzene (CDNB) in methanol, 25 µL distilled water and 12 µL of 5 mM reduced glutathione (GSH) was incubated at 30 °C for 5 min. Immediately before the absorbance measurement, 6.5 µL of the sample was added to the mixture. Absorbance was recorded at 340 nm at 0, 5, and 10 min. The results were expressed as U/mg of proteins.

#### 2.2.2. Proteolytic Enzymes

Trypsin activity was assayed using 20 mM N-α-benzoyldlarginine-p-nitroanilide (BAPNA) as the substrate, according to Bisswanger [25]. Trypsin (type II from porcine pancreas, EC 3.4.21.4) was used to generate the standard curve. Absorbance was measured at 405 nm, and the results were expressed as mU/mg proteins.

Chymotrypsin activity was determined following the protocol described by Hummel [26] and Wirnt [27]. N-Benzoyl-L-Tyrosine Ethyl Ester (BTEE; 1.18 mM) was used as the substrate. The reaction mixture, consisting of 80 mM Tris-HCl buffer, 100 mM calcium chloride (pH 7.8) and the sample, was monitored at 256 nm for 3 min. α-chymotrypsin was used to draw the standard curve. Trypsin and chymotrypsin activities were expressed as mU/mg proteins.

Aminopeptidase-N activity was assayed using 2.04 mM L-alanine-p-nitroanilide as the substrate, according to Liu and Wang [28] and del Valle and Mañanes [29]. The results were expressed as U/mg proteins.

Carboxypeptidase-like A and B activities were determined according to the protocol described by Grogan and Hunt [30]. For carboxypeptidase A activity, 1 mM Hippuryl-L-phenylalanine (HPA) dissolved in 25 mM Tris-HCl buffer (pH 7.5) containing 500 mM NaCl, was added to the sample. For carboxypeptidase B activity, 1 mM Hippuryl-L-arginine (HA) dissolved in 25 mM Tris HCl buffer (pH 7.65) containing 100 mM NaCl was added to the sample. Changes in absorbance at 254 nm for 2 min were recorded as an index of enzymatic activity for both enzymes. The results were expressed as U/mg of proteins and mU/mg proteins for carboxypeptidases A and B, respectively.

#### 2.2.3. Carbohydrases

Glucose oxidase activity was measured according to Schepartz and Subers [31] with some modifications. Briefly, a solution containing 3.5 M glucose in 0.2 M sodium phosphate buffer, pH 6.1, O-Dianisidine (3.5 mg/mL in 95% ethanol), horseradish peroxidase (HRP; 0.04 mg/mL in 0.2 M sodium phosphate buffer, pH 6.1) and the saliva sample was incubated at 37 °C for 15 min. Absorbance was measured at 405 nm. The values were expressed as mU/mg proteins.

Amylase activity was determined according to Hassanatabar and colleagues [32] with some modifications. Briefly, the sample was incubated with 0.5% rice starch in 0.1 M phosphate buffer (PB, pH 7) at 35 °C for 10 min. Then, 1% dinitro salicylic acid (DNS) in PB pH 7 was added to the mixture, which was subsequently boiled for 5 min. After cooling, water was added, and absorbance was measured at 540 nm. The values were expressed as mg of maltose produced/mg of proteins.

Cellulase activity was determined using carboxymethylcellulose (CMC) as the substrate, according to Gusakov and colleagues [33], and expressed as U/mg proteins. Absorbance was measured at 540 nm.

Lignin peroxidase activity was determined according to the protocol described by Gomes and colleagues [34] and expressed as µU/mg of proteins. Absorbance was measured at 310 nm for 3 min.

Chitinase activity was determined using chitin azure as the substrate according to Byrne and colleagues [35], with some modifications. First, 25 µL of the sample was incubated at 25 °C with 25 µL of chitin azure and 200 µL of 50 mM sodium acetate buffer, pH 5.0, for increasing periods of time (0, 1, 2, 3 and 6 min). Then, the reaction was stopped by adding 2 M HCl. The solution was cooled, and absorbance was measured at 550 nm. The values were expressed as ΔAbs/min/mg proteins.

α-Glycosidase activity was determined according to Mehrabadi and colleagues [36] using p-nitrophenyl-α-D-glucopyranoside as the substrate. Absorbance was measured at 405 nm for 10 min. The values were expressed as U/mg proteins.

#### 2.2.4. Esterases

Alkaline and acidic phosphatase activities were determined according to Binder and colleagues [37], and the values were expressed as mU/mg proteins.

Lipase activity was determined using a Lipase Assay kit (MAC482, Sigma-Aldrich). This assay is based on the dimercaptopropanol tributyrate (BALB) method, in which thiol (-SH) groups released by lipase-mediated cleavage of BALB react with 5,5′-dithiobis(2-nitrobenzoic acid) (DTNB) to form a colored product. The assay was performed according to the manufacturer’s instructions, and absorbance was measured at 412 nm. The values were expressed as mU/mg proteins.

Esterase activity was determined according to Tecles and colleagues [38], with some modifications. 4-Nitrophenol acetate was used as the substrate (5.5 mM) in 0.1 M phosphate saline buffer (PBS, pH 7.4). Absorbance was measured at 405 nm for 10 min. The values were expressed as U/mg proteins.

### 2.3. Statistical Analysis

Welfare assessment indicators were expressed as a single number, as indicated by the AWIN protocol. Enzyme activity data were reported for each enzyme as the mean, standard error, and minimum and maximum values of the fifteen ewes analyzed.

## 3. Results

Data from the sheep farm investigated were in line with the optimal welfare reference values reported in the AWIN test protocol (Table 2). The only exceptions were lamb survival, concerned with the characteristics of the farm, and the familiar approach to humans. The recorded lamb survival was 94%, whereas the familiar approach to humans was >5 m, without voluntary contact. Based on the stocking density, each adult ewe had 2.9 m^2^ of space available. The sheep had access to two automatic drinkers, two troughs, and two natural ponds, all containing clean and easily accessible water.

Esterase activity showed the highest value among the enzymes analyzed (12.95 ± 1.25 U/mg of proteins), whereas lignin peroxidase activity was the lowest (2.23 ± 0.37 µU/mg of proteins; Table 3). Glutathione S transferase and α-glycosidase activities were investigated but were not detected in the analyzed samples. In addition, the activities of catalase, chymotrypsin, carboxypeptidases A and B, chitinase, alkaline and acid phosphatases were found only in some of the samples analyzed.

Enzymatic activity data were also normalized to the volume of saliva used in the assays (Table 4). Normalization confirmed both a high esterase activity and a low lignin peroxidase activity (38.73 ± 3.28 U/mL; 6.43 ± 0.92 mU/L, respectively).

## 4. Discussion

Following the AWIN protocol, the flock’s welfare status was optimal for all parameters investigated, with the exception of lamb survival and the familiar human approach test. However, the lamb survival rate (94%) was better than that reported on farms in Europe (average 88.4%) [39]. This indicates that the farm’s conditions are optimal by European standards, demonstrating a high level of animal welfare. Regarding the familiar human approach test, behaviors such as escape attempts are typical expressions of fear, as observed in this investigation. A fear response towards humans may occur in extensively managed animals that are not used to being handled by strangers [40]. Therefore, assessment of this criterion may require different methods or different values placed on the same measure when considering extensively managed or housed sheep. It may be advisable to conduct familiar human approach tests according to the farm routine (e.g., during the milking session), as sheep are used to the farmer and remain calm, showing no fear in his presence [41]. It was also suggested that improving human–animal interactions by increasing farmers’ knowledge and awareness of animal stress and welfare could be beneficial [42].

Investigations into the enzymatic composition of ruminant saliva are very poor, likely due to the intrinsic complexity of rumen structure and function [5]. Due to rumination and the intense enzymatic activity of microorganisms present in the rumen, it is difficult to determine the origin of the enzymes detected in saliva. Diets based on a wide variety of natural foods may exert several effects beyond purely nutritional ones, including the modulation of salivation through neuroendocrine mechanisms [43,44]. Extensive animal husbandry relies on food sources available in natural pastures, allowing animals to freely consume a heterogeneous diet composed of diverse molecules. Changes in salivary protein composition have been observed in response to different feedstuffs and/or dietary substances [45,46,47]. Moreover, the selection of specific feed sources may promote the production of saliva types that are more efficiently adapted to those diets [47,48]. Bitter or astringent substances may trigger rapid and copious salivary secretion as an initial defense mechanism to dilute potentially harmful compounds introduced into the oral cavity, thereby alleviating these sensations [47]. One of the few studies examining multiple enzyme activities in the saliva of animals was conducted in the porcupine [49].

Among antioxidant enzymes, catalase activity was detected in sheep saliva, whereas glutathione S transferase activity was not found. Catalase has two enzymatic functions: it catalyzes the decomposition of H_2_O_2_ into O_2_ and H_2_O, and at low concentrations of H_2_O_2_, it catalyzes the oxidation of electron donors such as ethanol or phenols [50,51]. Iodide peroxidase has been purified from goat submaxillary glands; however, this enzyme is not present in sheep salivary glands [52]. Mahalingam and colleagues [53] reported that in humans, catalase originates from microorganisms. Because catalase activity was detected in only three of the fifteen analyzed samples, the origin of this enzyme cannot be determined without a proteomic approach. The function of glutathione S transferase is the detoxification of electrophiles (e.g., by-products of reactive oxygen species activity and polycyclic aromatic hydrocarbons) through conjugation with glutathione [54]. Glutathione S transferase activity was not detected in sheep saliva, whereas in porcupine saliva, it was reported at a value of 20.21SD16.62 mM/mg proteins [49]. Inducers of glutathione S transferase are abundant in vegetables such as those belonging to the Cruciferae and Liliaceae families [55,56], which are not part of the diet of the sheep investigated but are commonly included in the porcupine diet [57].

Regarding protein metabolism, five types of proteases were investigated: trypsin, chymotrypsin, N-aminopeptidase, and carboxypeptidases A and B. Proteolytic enzymes hydrolyze peptide bonds [58]. The secretion of saliva into the oral cavity exposes the proteins to a high number of proteolytic and other enzymes originating from oral microorganisms, epithelial cells and from leucocytes, which enter the mouth via the gingival crevice [46,59]. Mahalingam and colleagues [53] suggested that human proteinases and peptidases originate from microorganisms and leucocytes. Approximately 30–50% of ruminal microorganisms exhibit proteolytic activity [59]. The peptides and amino acids generated by extracellular ruminal proteolysis are transported into microbial cells, where they are further degraded by peptidases into amino acids. These amino acids can be incorporated into microbial protein or further deaminated to produce volatile fatty acids, CO_2_ and ammonia [59]. It can be assumed that some essential amino acids derived from the degradation of proteins in the rumen may be absorbed by sheep, although volatile fatty acids derived from starch and fiber constitute the main nutritional sources. Trypsin, chymotrypsin and N-aminopeptidase activities were lower than those reported in porcupine saliva [49]. This difference may be related to dietary habits, as crested porcupines occasionally consume carrion [60], whereas sheep do not. In sheep, the primary protein source originates from dead fungi, protozoa and bacteria within the rumen, and the products of their digestion may be utilized by other ruminal microorganisms for their survival [59]. Carboxypeptidase A activity was higher than that of carboxypeptidase B; however, neither enzymatic activity was detected in all saliva samples analyzed (11/15 and 13/15, respectively). To the best of our knowledge, the origin of these two enzymes remains unclear without a proteomic approach. Among carbohydrases, in this preliminary investigation, a high level of glucose oxidase activity was detected. Glucose oxidase catalyzes the oxidation of D-glucose to gluconic acid, using molecular oxygen as an electron acceptor with simultaneous production of hydrogen peroxide [61]. Glucose oxidase has been identified as an extracellular enzyme in fungi, as a membrane-binding protein in Gram-negative bacteria, and as a cytosolic enzyme in some insects [62]. As this enzyme is not considered to be of sheep origin, the high variability observed in this study may be due to interindividual differences in ruminal microbial communities. Amylase and cellulase hydrolyze starch and cellulose, the first a reserve polysaccharide and the second a structural component of plant cells, both of which are essential components in the diet of ruminants [63]. Amylase is the major salivary enzyme in animals. Data obtained in this investigation demonstrated the presence of amylase activity in sheep saliva. The sheep examined received two daily rations of oat seeds during milking, and saliva samples were taken within two hours of this process.

The sheep used in this investigation were fed a diet that also contained starch, justifying the presence of the activity of this enzyme. This feeding regime may account for the high amylase activity observed. Several authors have reported the absence of salivary amylase in the sheep proteome [46,64,65], suggesting that this may be related to a lack of starch in the diet. Nevertheless, Fuentes-Rubio and colleagues [20] and Contreras-Aguilar and colleagues [14] reported amylase activity in the saliva of sheep, with Contreras Aguilar and colleagues [14] further investigating it as a biomarker of stress. This study also included amylase to assess its activity under verified sheep welfare conditions, according to the AWIN protocol. As amylase is not detected in the sheep saliva proteome, it is assumed to originate from the rumen microbiota (bacteria, fungi or protozoa). In elephants, amylase activity decreases after feeding [66], which may explain the great variability observed in this study (4.61–60.91 mg of maltose produced/mg of proteins). Cellulase activity was detected in all sheep saliva samples. While the presence of symbiotic protozoa or bacteria has been used to explain cellulose digestion in herbivores such as cattle, Watanabe and Tokuda [67] also described the presence of cellulases of animal origin (insects and crayfish). Therefore, in this investigation, cellulase activity may originate both from the sheep and from ruminal microorganisms. The results obtained in this investigation showed that lignin peroxidase and chitinase activities were also present in sheep saliva. Lignin peroxidases catalyze hydrogen peroxide-mediated oxidative lignin degradation [68], whereas chitinases catalyze the hydrolytic cleavage of the β-1,4-glycoside bond present in biopolymers of N-acetylglucosamine, such as chitin [69]. The origin of lignin peroxidase is unknown, but it could be produced by organisms present in the oral cavity, for example, fungi [34]. Van Steijn and colleagues [70] suggested that mammalian chitinases serve as a defense mechanism against invasion by chitin-containing pathogens. Furthermore, it has been hypothesized that insectivorous bat salivary lysozyme could serve as a chitinase, whereas in human saliva, lysozyme and chitinase are clearly distinct enzymes [70]. Indeed, Kasprzewska [69] reported chitinases in various organisms, including bacteria, yeast and fungi. To expand our knowledge of lignin peroxidase and chitinase activities and their functionality in sheep, a proteomic approach is highly desirable. Alpha-glycosidases catalyze the hydrolysis of α-glucosidic linkages at the non-reducing ends of substrates to release α-glucose [71]. Alpha-glycosidase (maltase) activity was not detected in this investigation, which may be due to the absence of malt in the diets of sheep, as malt can be added through by-products of alcohol production, leading to the presence of maltase in saliva [72]. Mahalingam and colleagues [53] reported that, in humans, maltase originates from microorganisms and leucocytes, and it can be assumed that in ruminants, it derives from microbial flora.

Esterases catalyze the first step of ester bond hydrolysis [73]. The results obtained in this study detected both acidic and alkaline phosphatase among the esterase activities present in sheep saliva. In sheep, the alkaline phosphatase is involved in ionic movement across the cell membrane [74]. It is abundantly expressed in the endothelium of small blood vessels and in the cells specialized for endocytosis and pinocytosis [74]. In sheep, a moderate concentration of acid phosphatase has also been reported in parotid and submandibular saliva, whereas marked alkaline phosphatase activity was found in submaxillary fluid [11,65]. Furthermore, Mahalingam and colleagues [53] suggested that in humans, both acidic and alkaline phosphatases originate from glands, microorganisms and leucocytes. The lipase activity observed in this investigation is consistent with the findings of Contreras Aguilar and colleagues [14]. In young ruminants, palatine salivary glands (*glandulae veli palatini*) are responsible for the secretion of salivary lipase [74]. Mahalingam and colleagues [53] also detected lipases originating from microorganisms and leucocytes in humans. The esterase activity measured in the present investigation was higher than that reported by Contreras Aguilar and colleagues [14]. Esterase presence was already observed in the parotid salivary gland of prenatal sheep [75], while in adult sheep, esterases have been reported at moderate concentrations in both parotid saliva and mandibular saliva [76]. After investigating lipase and esterase as new biomarkers of stress in the saliva of sheep, Contreras Aguilar and colleagues [14] reported that only lipase showed the greatest increase after stressful stimuli. This study also involved these two enzymes to assess their activity in a condition of verified sheep welfare, in accordance with the AWIN protocol.

## 5. Conclusions

This study reported and comprehensively screened enzymatic activities in sheep saliva involving a broad range of enzymes. Catalase, trypsin, chymotrypsin, N-aminopeptidases, both carboxypeptidases A and B, glucose oxidase, amylase, cellulase, lignin peroxidase, chitinase, both alkaline and acidic phosphatases, lipase, and esterase activities were detected in most of the sheep saliva samples analyzed. Only two enzymatic activities, glutathione S transferase and α-glycosidase, were investigated but not detected. In conclusion, this investigation provides information on salivary enzymes of sheep farmed under verified welfare conditions. This information could serve as a useful tool for future investigations into the adaptive response of sheep to diet and different environmental stressors, potentially contributing to the assessment of health and nutritional status in this species. Furthermore, the sample collection protocol used in this investigation has advantages for both the operator and the animal. In fact, ruminant saliva is abundant and can be easily collected by the Salivette^®^ device without requiring specialized staff. The selected farm was a small, family-run business with a limited number of animals; nevertheless, the farm conditions had a high degree of welfare related to the European mean. Further investigations on the origin of individual enzymes, using a proteomic approach, are desirable to distinguish between enzymes that originate from microorganisms and those produced by sheep salivary glands.

## Figures and Tables

**Table 1:** Welfare assessment according to the questionnaires of the AWIN protocol for sheep.

Parameters	Scores
**Stereotypy**	Percentage of ewes without stereotypic behavior (20 min of observation)
**Social withdrawal**	Percentage of ewes without social withdrawal (20 min of observation)
**Excessive itching**	Percentage without excessive itching or scratching (20 min of observation)
**Panting**	Percentage of ewes with normal respiration
**Fleece cleanliness**	Score of ewes with clean fleece (scores 0 and 2)
**Fleece quality**	Percentage of ewes with good fleece coverage
**Tail length**	Percentage of ewes with full tails added to ewes with docked tails of an adequate length
**Fecal soiling**	Score 0 of ewes with an acceptably clean breech area (scores 0, 1, and 2)
**Lameness**	Score of ewes that are not lame (scores 0 and 1)
**Familiar human approach test**	Closest distance of approach of human to sheep (<5 m) Sheep initiated voluntary contact (Y/N)
**Stocking density**	100% if assessed pens provide good space availability
**Water availability**	From 100% if all animals have access to a clean and adequate water supply to 0% if water supply is inadequate and dirty

**Table 2:** Welfare assessment of the sheep farm object of this investigation, following the AWIN protocol.

Indicator	Data Recorded	Optimal Data
Lamb survival	94%	100%
Stereotypy	0%	0%
Social withdrawal	0%	0%
Excessive itching	0%	0%
Panting	0%	0%
Fleece cleanliness	0	0
Fleece quality	100%	100%
Tail length	100%	100%
Fecal soiling	0	0
Lameness	0	0
Familiar human approach test	>5 m, without voluntary contact	voluntary contact
Stocking density	100%	100%
Water availability	100%	100%

**Table 3:** Enzymatic assays on saliva samples (n = 15) of sheep (calculated on saliva protein content) grouped based on the type of substrate. For each enzyme, the mean, SE, minimum (Min) and maximum (Max) values are reported.

Enzymatic Activity	Mean (SE)	Min	Max	n of Samples in Which Enzyme Activity Was Present
**Antioxidant enzymes**				
Catalase (U/mg of proteins)	0.81 (0.60)	0	8.74	3
Glutathione S transferase (U/mg of proteins)	nd	nd	nd	nd
**Proteolytic enzymes**				
Trypsin (mU/mg of proteins)	0.07 (0.02)	0.01	0.18	15
Chymotrypsin (mU/mg of proteins)	0.34 (0.10)	0	1.67	14
Aminopeptidase (U/mg of proteins)	0.79 (0.25)	0.13	3.74	15
Carboxypeptidase A (U/mg of proteins)	0.11 (0.07)	0	1.02	11
Carboxypeptidase B (mU/mg of proteins)	39.87 (8.69)	0	97.27	13
**Carbohydrases**				
Glucose oxidase (mU/mg of proteins)	326.26 (74.71)	33.12	874.69	15
Amylase (mg of maltose produced/mg of proteins)	26.97 (5.88)	4.61	60.91	15
Cellulase (U/mg proteins)	2.72 (0.11)	1.97	3.45	15
Lignin peroxidase (µU/mg of proteins)	2.23 (0.37)	0.71	4.60	15
Chitinase (ΔAbs/min/mg proteins)	0.29 (0.10)	0	1.09	13
α-glycosidase (U/mg of proteins)	nd	nd	nd	nd
**Esterases**				
Alkaline phosphatase (mU/mg of proteins)	5.47 (1.59)	0	20.75	12
Acid phosphatase (mU/mg of proteins)	6.22 (2.22)	0	24.78	13
Lipase (mU/mg of proteins)	16.66 (4.05)	1.79	48.42	15
Esterase (U/mg of proteins)	12.95 (1.25)	5.84	21.42	15

nd = not detected.

**Table 4:** Enzymatic assays on saliva samples of sheep (n = 15) calculated based on saliva volume and grouped based on the type of substrate. For each enzyme, the mean, SE, minimum (Min) and maximum (Max) values are reported.

Enzymatic Activity	Mean (SE)	Min	Max
**Antioxidant enzymes**			
Catalase (U/mL)	2.66 (1.95)	0	28.63
Glutathione S transferase (U/L)	nd	nd	nd
**Proteolytic enzymes**			
Trypsin (U/L)	0.20 (0.04)	0.03	0.59
Chymotrypsin (U/L)	1.06 (0.31)	0	5.09
Aminopeptidase (U/mL)	2.17 (0.60)	0.41	8.86
Carboxypeptidase A (U/mL)	0.40 (0.27)	0	4.20
Carboxypeptidase B (U/L)	117.33 (23.56)	0	296
**Carbohydrases**			
Glucose oxidase (U/L)	1021 (235)	108	2338
Amylase (g of maltose produced/L)	79.26 (17.37)	15.30	225.74
Cellulase (U/mL)	8.20 (0.29)	7.04	11.30
Lignin peroxidase (mU/L)	6.43 (0.92)	2.26	13.55
Chitinase (ΔAbs/min/mL)	0.92 (0.31)	0	2.69
α-glycosidase (U/L)	nd	nd	nd
**Esterases**			
Alkaline phosphatase (U/L)	17.79 (5.91)	0	82.66
Acid phosphatase (U/L)	17.04 (6.03)	0	75.41
Lipase (U/L)	48.46 (10.76)	5.10	121.48
Esterase (U/mL)	38.73 (3.28)	19.00	61.00

nd = not detected.

## Data Availability

The data presented in this study are available from the corresponding author upon reasonable request.

## Abbreviations

The following abbreviations are used in this manuscript:

AAOPs	Advanced oxidation protein products
FRAP	Ferric reducing ability of plasma
CUPRAC	Cupric reducing antioxidant capacity
TEAC	Trolox equivalent antioxidant capacity
CDNB	1-chloro-2,4-dinitrobenzene
BAPNA	N-α-benzoyldlarginine-p-nitroanilide
BTEE	N-Benzoyl-L-Tyrosine Ethyl Ester
HPA	Hippuryl-L-phenylalanine
HA	Hippuryl-L-arginine
HRP	Horseradish peroxidase
PB	Phosphate buffer
DNS	Dinitro salicylic acid
CMC	Carboxymethylcellulose
BALB	Dimercaptopropanol tributyrate
